# Influence of Implant Positioning on Peri-Implant Colonization by Porphyromonas gingivalis and Fusobacterium nucleatum: A Prospective Longitudinal Observational Cohort Study

**DOI:** 10.7759/cureus.112829

**Published:** 2026-07-16

**Authors:** Reena Pabri, Garima Sharma, Ghata Savoriya, Meenakshi Daiya, Samir Mansuri, Pallavi Digambar Dashatwar

**Affiliations:** 1 Department of Oral Pathology and Microbiology, College of Dental Sciences, Rajasthan University of Health Sciences (RUHS), Jaipur, IND; 2 Department of Prosthodontics, College of Dental Sciences, Rajasthan University of Health Sciences (RUHS), Jaipur, IND; 3 Department of Oral and Maxillofacial Surgery, Al Kuwait Hospital, Dubai, ARE; 4 Department of Oral Pathology and Microbiology, Saraswati Dhanwantari Dental College and Hospital, Parbhani, IND

**Keywords:** dental implants, fusobacterium nucleatum, peri-implantitis, polymerase chain reaction, porphyromonas gingivalis

## Abstract

Introduction: Peri-implant microbial colonization plays a critical role in the maintenance of peri-implant tissue health and the development of peri-implant diseases. Implant positioning relative to crestal bone may influence the peri-implant microenvironment and affect the accumulation of pathogenic microorganisms. However, limited evidence is available regarding the relationship between implant placement depth and the prevalence of key periodontal pathogens. The present study aimed to evaluate and compare the prevalence and quantitative levels of *Porphyromonas gingivalis* and *Fusobacterium nucleatum* in the peri-implant region of subcrestal and supracrestal dental implants, and to assess their association with peri-implant clinical parameters.

Materials and methods: This prospective longitudinal observational cohort study included 40 participants with functional dental implants, including 20 subcrestal and 20 supracrestal implants. Peri-implant clinical parameters, including probing pocket depth (PPD), bleeding on probing (BOP), plaque index, and radiographic bone loss, were recorded. Peri-implant crevicular fluid samples were collected from sterile paper points and subjected to genomic DNA extraction. Quantitative real-time polymerase chain reaction (qPCR) was performed to detect and quantify *P. gingivalis* and *F. nucleatum*. Detection rates, bacterial loads, and correlations with clinical parameters were analyzed at a significance level of p < 0.05.

Results: Baseline demographic and clinical characteristics were comparable between the subcrestal and supracrestal implant groups, with no significant differences in age, sex, smoking status, implant location, PPD, BOP, plaque index, or radiographic bone loss (all p > 0.05). At 12 months, *P. gingivalis* was detected in 10 (50.0%) of supracrestal implants compared with 5 (25.0%) of subcrestal implants (p = 0.102), while *F. nucleatum* was detected in 13 (65.0%) and 7 (35.0%) of implants, respectively (p = 0.058). Quantitative analysis demonstrated significantly higher bacterial loads in supracrestal implants for both *P. gingivalis* (1.95 ± 0.86 vs. 1.52 ± 0.79 log₁₀ copies; p = 0.042) and *F. nucleatum* (2.32 ± 0.91 vs. 1.89 ± 0.84 log₁₀ copies; p = 0.031). In the supracrestal group, bacterial loads showed stronger positive correlations with peri-implant clinical parameters, with the strongest association observed between *F. nucleatum* and PPD (r = 0.61, p = 0.005), followed by BOP (r = 0.58, p = 0.007) and radiographic bone loss (r = 0.55, p = 0.009).

Conclusions: Supracrestal implant placement is associated with increased colonization by periodontal pathogens and stronger relationships between bacterial burden and peri-implant tissue deterioration. Subcrestal implant placement may provide a more favorable microbiological environment and contribute to improved peri-implant tissue health.

## Introduction

Dental implants have become a predictable and widely accepted treatment modality for the replacement of missing teeth, demonstrating high survival rates and favorable functional outcomes [1,2]. However, long-term implant success is influenced by multiple biological and mechanical factors, among which peri-implant microbial colonization plays a critical role. The establishment of a pathogenic biofilm around implant surfaces may initiate inflammatory changes in peri-implant tissues, leading to peri-implant mucositis and peri-implantitis, which can compromise implant stability and longevity [3]. The development of peri-implantitis is influenced by complex interactions between pathogenic microorganisms and the host immune response. Bhukal et al. [4] reported significant associations between proinflammatory and anti-inflammatory cytokines and peri-implantitis, emphasizing the biological relevance of microbial colonization in initiating peri-implant tissue inflammation.

Among the microorganisms implicated in peri-implant disease, *Porphyromonas gingivalis* and *Fusobacterium nucleatum* are key periodontal pathogens [5]. *P. gingivalis *is a gram-negative anaerobic bacterium associated with tissue destruction and immune dysregulation, whereas* F. nucleatum* functions as a bridging organism that facilitates the maturation and complexity of microbial biofilms [6]. The presence and abundance of these species have been linked to increased peri-implant inflammation, probing depth, bleeding on probing (BOP), and marginal bone loss.

Implant positioning relative to the crestal bone may influence the peri-implant environment and microbial colonization patterns. Subcrestal implant placement has been advocated for improved esthetics and maintenance of peri-implant tissues, whereas supracrestal placement may alter the microenvironment at the implant-abutment interface, potentially affecting bacterial accumulation and peri-implant tissue health [7]. A more coronally positioned microgap may facilitate bacterial ingress and biofilm accumulation, whereas subcrestal placement may provide better soft tissue adaptation and protection against microbial penetration. Despite these biologically plausible mechanisms, clinical evidence regarding the influence of implant positioning on peri-implant pathogens remains limited.

Therefore, the present prospective longitudinal observational cohort study aimed to evaluate and compare the microbial presence and quantitative levels of *P. gingivalis* and *F. nucleatum* in the peri-implant region of patients with subcrestal and supracrestal dental implants using quantitative real-time polymerase chain reaction (qPCR) analysis. Additionally, this study aimed to assess the relationship between bacterial loads and clinical peri-implant parameters, including probing pocket depth (PPD), BOP, and radiographic bone loss, to better understand the microbiological implications of implant positioning.

## Materials and methods

Study design and setting

This prospective longitudinal observational cohort study was conducted in the Department of Prosthodontics, College of Dental Sciences, Rajasthan University of Health Sciences (RUHS), Jaipur, India, in collaboration with the Department of Oral Pathology and Microbiology. Participant recruitment was conducted between February 2025 and April 2025, and all enrolled participants were followed prospectively until April 2026. Ethical approval was obtained from the Institutional Ethics Committee of the College of Dental Sciences before study commencement (approval no.: RUHS/CDS/IEC/2025/02/12). Written informed consent was obtained from all participants before enrollment. All dental implants included in the study had been placed previously as part of routine clinical treatment by experienced implant surgeons according to individual patient requirements and the treating clinician's treatment plan. The investigators did not influence implant placement depth or surgical procedures. Eligible participants were enrolled during routine implant maintenance visits after their implants had been in functional loading for at least six months following definitive prosthesis delivery.

Sample size estimation

The sample size was calculated using the G*Power software version 3.1 (Heinrich Heine University Düsseldorf, Düsseldorf, Germany). Based on a previously published study evaluating microbial diversity in peri-implant tissues, an effect size of 0.85 was considered for differences in bacterial load between the study groups [8]. Considering a statistical power of 80%, a confidence level of 95%, and a two-tailed test, the minimum required sample size was estimated to be 36 participants. To compensate for possible attrition and improve statistical reliability, 40 participants were recruited, comprising 20 patients with subcrestal implants and 20 patients with supracrestal implants.

Participant selection and grouping

Adult patients aged ≥18 years attending routine implant follow-up visits were screened for eligibility. Individuals with at least one functional screw-retained or cement-retained dental implant that had been in function for a minimum of six months following definitive prosthesis delivery were considered eligible. Radiographic records and surgical documentation were reviewed to determine implant position. Based on these records, implants were categorized into two groups. Group A consisted of subcrestal implants, defined as implants placed approximately 1-2 mm apical to the alveolar crest at the time of surgery [7]. Group B consisted of supracrestal implants, defined as implants positioned at or slightly above the crestal bone level. To avoid clustering effects, only one implant site per participant was included in the study.

Inclusion and exclusion criteria

Participants who were systemically healthy or had medically controlled systemic conditions and possessed adequate radiographic and surgical documentation to confirm implant position were included. Individuals who had received periodontal or implant therapy within the previous three months were excluded. Additional exclusion criteria included uncontrolled systemic diseases, such as uncontrolled diabetes mellitus; immunosuppressive disorders; antibiotic therapy or antiseptic mouth rinse use during the preceding three months, pregnancy or lactation, implant mobility or loss of osseointegration, and orthodontic treatment within the previous six months.

Participant timeline and sampling schedule

Eligible participants were enrolled during routine implant maintenance visits after their implants had been in functional loading for at least six months following definitive prosthesis delivery. The enrollment visit served as the baseline study assessment (T0). At this visit, peri-implant clinical parameters were recorded, and peri-implant crevicular fluid (PICF) samples were collected for microbiological analysis. Participants were subsequently followed prospectively, with identical clinical examinations and microbiological sampling repeated at 6 months (T1) and 12 months (T2) after the baseline study visit. All microbiological assessments were performed prospectively according to this predefined follow-up schedule using the same standardized sampling and laboratory protocols.

Clinical and radiographic assessment

All clinical examinations were performed by a calibrated examiner under standardized conditions. The peri-implant PPD was recorded in millimeters at four to six sites around each implant using a plastic periodontal probe. BOP was assessed and recorded as present or absent, and the cumulative BOP score was calculated based on the number of bleeding sites. Plaque accumulation was evaluated using the plaque index [9]. Radiographic assessment was performed using standardized periapical radiographs obtained using the long-cone paralleling technique. Marginal bone loss was measured as the distance from the implant shoulder to the first bone-to-implant contact and was recorded in millimeters. The implant position was reconfirmed radiographically before inclusion in the study groups. All clinical and radiographic measurements were performed by a single calibrated examiner. Examiner calibration was conducted before the study using repeated measurements on 10 implant sites not included in the final analysis, yielding an intraclass correlation coefficient (ICC) of >0.90, indicating excellent intra-examiner reliability.

Collection of PICF samples

PICF and subgingival biofilm samples were collected using a standardized protocol. The implant region was isolated using sterile cotton rolls and gently air-dried to eliminate salivary contaminants. Supragingival plaque deposits were carefully removed using a sterile scaler, without disturbing the subgingival environment. Sterile Periopaper® collection strips (OraFlow; Smithtown, NY, USA) were inserted into the deepest peri-implant sulcus or pocket and maintained in position for 30 seconds [10]. Following sample collection, the paper points were immediately transferred into sterile microcentrifuge tubes containing 500 μL of phosphate-buffered saline and stored at -80°C until laboratory analysis.

DNA extraction

Genomic DNA was extracted from the collected samples using a DNeasy Blood and Tissue Kit (Qiagen GmbH; Hilden, Germany) according to the manufacturer’s instructions [11]. Briefly, the bacterial cells were subjected to enzymatic and mechanical lysis using lysozyme and proteinase K. The released DNA was subsequently bound to silica membrane spin columns, washed to eliminate contaminants, and eluted in 50 μL of elution buffer. The DNA concentration and purity were assessed spectrophotometrically by measuring the absorbance ratio at 260/280 nm.

Quantitative polymerase chain reaction analysis

q-PCR was performed using SYBR Green chemistry on a CFX96 Real-Time PCR Detection System (Bio-Rad Laboratories; Hercules, CA, USA). Species-specific primers targeting the 16S rRNA genes of *P. gingivalis* and *F. nucleatum* were used for amplification. Each reaction mixture contained SYBR Green Master Mix, forward and reverse primers, template DNA, and nuclease-free water in a total reaction volume of 25 μL [12,13].

Thermal cycling conditions consisted of an initial denaturation step at 95°C for 5 min, followed by 40 amplification cycles of denaturation at 95°C for 15 s, annealing at 60°C for 30 s, and extension at 72°C for 30 s. A final extension step was performed at 72°C for 5 min, followed by melting curve analysis to confirm amplification specificity.

Reference strains of *P. gingivalis *(ATCC 33277; American Type Culture Collection, Manassas, VA, USA) and *F. nucleatum* (ATCC 25586; American Type Culture Collection, Manassas, VA, USA) served as positive controls. Reactions without template DNA were used as negative controls. Standard curves were generated using serial dilutions of known bacterial DNA concentrations to enable absolute quantification of the bacterial load.

Outcome measures

The primary outcome measures included detection rates and quantitative bacterial loads of *P. gingivalis* and *F. nucleatum* in the subcrestal and supracrestal implant groups. Detection rates are expressed as the presence or absence of bacterial species, whereas quantitative measurements are reported as log10-transformed bacterial copy numbers. Secondary outcome measures included the correlation between bacterial load and peri-implant clinical parameters, such as PPD, BOP, plaque index, and radiographic bone loss. To minimize potential confounding, baseline demographic and clinical characteristics, including age, sex, smoking status, implant location, duration of implant function, plaque index, and systemic health status, were recorded and compared between the study groups. Standardized inclusion and exclusion criteria were applied to reduce the influence of factors known to affect peri-implant microbial colonization.

Statistical analysis

Statistical analysis was performed using IBM SPSS Statistics for Windows, Version 25.0 (released 2017; IBM Corp., Armonk, NY, USA). Descriptive statistics are presented as mean ± standard deviation, median (range), or frequency and percentage, as appropriate. Data were analyzed for normal distribution with the Shapiro-Wilk test. Detection rates of bacterial species between the groups were compared using the chi-square test. Quantitative bacterial loads were analyzed using the Mann-Whitney U test because of the non-normal distribution of the data. Correlations between bacterial loads and clinical parameters were assessed using Spearman's rank correlation coefficient. Statistical significance was established at p < 0.05.

## Results

A total of 40 participants were included in the study, with 20 (50.0%) in the subcrestal implant group and 20 (50.0%) in the supracrestal implant group. The overall study population comprised 25 males (62.5%) and 15 females (37.5%). The baseline demographic and clinical characteristics were comparable between the two groups, with no statistically significant differences observed for age, sex distribution, smoking status, implant location, duration of implant function, PPD, BOP, plaque index, or radiographic bone loss (Table 1).

**Table 1:** Demographic and baseline clinical characteristics of the study participants. Data are presented as mean ± standard deviation, median (range), or frequency (percentage). An independent-samples t-test was used for age and radiographic bone loss. The Mann-Whitney U test was used for the duration of implant function, probing pocket depth, bleeding on probing score, and plaque index. Chi-square test was used for sex, smoking status, implant location, and bleeding on probing status. p < 0.05 was considered statistically significant. PPD: probing pocket depth, BOP: bleeding on probing; SD: standard deviation

Parameter		Subcrestal implants (n = 20)	Supracrestal implants (n = 20)	Total (n = 40)	Test statistics	p-value
Age (years)	Mean ± SD	42.3 ± 8.5	43.8 ± 9.2	43.1 ± 8.9	t = -0.612	0.542
Sex	Male	12 (60.0%)	13 (65.0%)	25 (62.5%)	χ² = 0.128	0.724
	Female	8 (40.0%)	7 (35.0%)	15 (37.5%)		
Smoking status	Non-smoker	17 (85.0%)	16 (80.0%)	33 (82.5%)	χ² = 0.248	0.621
	Smoker	3 (15.0%)	4 (20.0%)	7 (17.5%)		
Implant location	Maxilla	9 (45.0%)	10 (50.0%)	19 (47.5%)	χ² = 0.128	0.724
	Mandible	11 (55.0%)	10 (50.0%)	21 (52.5%)		
Duration of implant in function (months)	Median (range)	17 (12-30)	18 (12-32)	18 (12-32)	U = 188.00	0.732
Probing pocket depth (PPD), mm	Median (range)	2.4 (1.5-3.5)	2.5 (1.6-3.8)	2.5 (1.5-3.8)	U = 192.00	0.684
Bleeding on probing (BOP)	Positive (sites with BOP)	4 (20.0%)	5 (25.0%)	9 (22.5%)	χ² = 0.128	0.724
	Negative	16 (80.0%)	15 (75.0%)	31 (77.5%)		
BOP score (0-4)	Median (range)	0.3 (0-1)	0.4 (0-1.2)	0.4 (0-1.2)	U = 186.50	0.612
Radiographic bone loss, mm	Mean ± SD	0.18 ± 0.12	0.21 ± 0.14	0.19 ± 0.13	t = -0.782	0.442
Plaque index (0-3)	Median (range)	0.5 (0-1.2)	0.6 (0-1.4)	0.55 (0-1.4)	U = 189.00	0.558

Microbiological analysis demonstrated a progressive increase in the detection rates of *P. gingivalis* and *F. nucleatum* over the observation period in both groups. At baseline and six months, no significant differences were observed between the subcrestal and supracrestal implants. However, at 12 months, *P. gingivalis* was detected more frequently in the supracrestal implant group than in the subcrestal implant group, whereas *F. nucleatum *showed a trend that approached statistical significance (Table 2).

**Table 2:** Detection rate of Porphyromonas gingivalis and Fusobacterium nucleatum according to implant position and time point. Data are presented as frequency (percentage). Between-group comparisons were performed using the chi-square test, and p < 0.05 was considered statistically significant.

Time point	Implant position	*P. gingivalis* positive, n (%)	Test statistics	p-value	*F. nucleatum *positive, n (%)	Test statistics	p-value
Baseline	Subcrestal	3 (15)	0.17	0.677	5 (25)	χ² = 0.47	0.490
	Supracrestal	4 (20)			7 (35)		
6 months	Subcrestal	4 (20)	1.12	0.288	6 (30)	χ² = 1.66	0.197
	Supracrestal	7 (35)			10 (50)		
12 months	Subcrestal	5 (25)	2.66	0.102	7 (35)	χ² = 3.60	0.058
	Supracrestal	10 (50)			13 (65)		

Quantitative assessment of the bacterial load revealed increasing levels of both microorganisms over time in both implant groups. Although bacterial counts were comparable at baseline and 6 months, supracrestal implants exhibited significantly greater loads of both *P. gingivalis* and *F. nucleatum* at 12 months than subcrestal implants (Table 3).

**Table 3:** Quantitative levels of Porphyromonas gingivalis and Fusobacterium nucleatum according to implant position and time point. Data are presented as mean ± standard deviation and median of log₁₀-transformed bacterial copy numbers (copies/sample). Between-group comparisons were performed using the Mann-Whitney U test. Test values represent U statistics, and *p < 0.05 indicates statistical significance. SD: standard deviation

Time point	Implant position	*P. gingivalis*, mean ± SD (log₁₀)	Median (log₁₀)	Test statistics	p-value	*F. nucleatum*, mean ± SD (log₁₀)	Median (log₁₀)	Test statistics	p-value
Baseline	Subcrestal	1.12 ± 0.68	1.05	158	0.542	1.48 ± 0.72	1.42	152	0.418
	Supracrestal	1.25 ± 0.71	1.18			1.63 ± 0.75	1.57		
6 months	Subcrestal	1.35 ± 0.74	1.30	122	0.128	1.70 ± 0.79	1.65	118	0.105
	Supracrestal	1.68 ± 0.82	1.62			2.05 ± 0.88	2.00		
12 months	Subcrestal	1.52 ± 0.79	1.48	106	0.042*	1.89 ± 0.84	1.84	98	0.031*
	Supracrestal	1.95 ± 0.86	1.90			2.32 ± 0.91	2.27		

Correlation analysis demonstrated positive associations between the bacterial load and peri-implant clinical parameters. In the subcrestal implant group, *F. nucleatum* showed a significant correlation with PPD, whereas other correlations did not reach statistical significance. In contrast, the supracrestal implant group exhibited stronger and statistically significant positive correlations between bacterial species and clinical indicators of peri-implant inflammation and tissue breakdown. The strongest association was observed between *F. nucleatum* levels and PPD, followed by BOP, and radiographic bone loss (Table 4). Overall, the findings suggest that supracrestal implant positioning may favor greater colonization by periodontal pathogens and demonstrate stronger associations between bacterial burden and adverse peri-implant clinical parameters than subcrestal implant placement.

**Table 4:** Correlation between bacterial loads and peri-implant clinical parameters according to implant position. Data are presented as Spearman’s correlation coefficient (r) and corresponding p-value. Correlations were analyzed using Spearman’s rank correlation test; p < 0.05 was considered statistically significant. PPD: probing pocket depth, BOP: bleeding on probing

Clinical parameter	Subcrestal: *P. gingivalis*		Subcrestal: *F. nucleatum*		Supracrestal: *P. gingivalis*		Supracrestal: *F. nucleatum*	
	r-value	p-value	r-value	p-value	r value	p-value	r-value	p-value
PPD	0.31	0.184	0.45	0.047	0.52	0.018	0.61	0.005
BOP	0.28	0.221	0.39	0.083	0.47	0.032	0.58	0.007
Bone loss	0.22	0.339	0.33	0.149	0.49	0.027	0.55	0.009

## Discussion

The present prospective longitudinal observational cohort study evaluated the prevalence and quantitative levels of *P. gingivalis* and* F. nucleatum* in the peri-implant region of subcrestal and supracrestal dental implants, and examined their association with peri-implant clinical parameters. The findings demonstrated that although both implant groups exhibited progressive microbial colonization over time, supracrestal implants showed higher detection rates and significantly greater bacterial loads at 12 months. Furthermore, the bacterial burden exhibited stronger correlations with PPD, BOP, and radiographic bone loss in the supracrestal implant group.

The baseline comparability of demographic and clinical variables between the groups strengthens the validity of the findings by reducing the influence of confounding factors. No significant differences were observed in age, sex distribution, smoking status, implant location, plaque accumulation, or peri-implant clinical parameters at baseline, indicating that the observed microbiological differences were more likely attributable to implant positioning than participant-related characteristics. Similar methodological approaches have been adopted in peri-implant microbiological studies to ensure comparability between implant groups and to minimize selection bias.

A progressive increase in the detection rates of *P. gingivalis* and *F. nucleatum* was observed throughout the study. These findings are biologically plausible because microbial colonization of implant surfaces begins immediately after implant exposure to the oral environment and gradually evolves into a mature biofilm [7]. *F. nucleatum *serves as a bridging microorganism facilitating the co-aggregation of early and late colonizers, whereas *P. gingivalis* is considered a keystone pathogen capable of disrupting host-microbial homeostasis and promoting chronic inflammation [10,14]. The increasing prevalence of these microorganisms over time is consistent with the observations reported by Galvão et al. [8], who demonstrated substantial microbial diversity and increased colonization by periodontopathogenic species in peri-implant environments.

The significantly higher detection frequency of *P. gingivalis* and the trend toward greater detection of *F. nucleatum* in supracrestal implants at 12 months suggest that implant positioning may influence the microbial ecology. Supracrestal implant placement may expose the implant-abutment microgap closer to the oral environment, potentially facilitating bacterial ingress and biofilm accumulation. Previous investigations by Canullo et al. [15] and Romanos and Weitz [16] have suggested that implant positioning and the implant-abutment interface can affect peri-implant tissue stability and microbial colonization patterns. Although these studies have primarily focused on peri-implant tissue health and crestal bone changes, their findings support the concept that implant design and positioning influence the local biological environment.

q-PCR analysis revealed significantly greater loads of both pathogens in the supracrestal implants at 12 months. This observation corroborates previous reports demonstrating a relationship between increased pathogenic bacterial counts and peri-implant inflammation [17]. Kumar et al. [18] reported that peri-implant disease sites harbor significantly greater levels of anaerobic periodontal pathogens, particularly *P. gingivalis* and *F. nucleatum*, than healthy implant sites. Similarly, Lafaurie et al. [19] demonstrated that these microorganisms were among the predominant species associated with peri-implant disease progression. Therefore, the higher bacterial burden observed around supracrestal implants may indicate a greater susceptibility to biological complications if preventive measures are not implemented.

The present findings are supported by previous investigations that evaluated the influence of implant positioning and microbial colonization around dental implants. de Moraes Rego et al. [20] reported significantly higher counts of specific bacterial species around implants placed at the supracrestal level than those positioned at the crestal level, suggesting that implant placement depth may influence the peri-implant microbial environment. Similarly, Silva-Boghossian et al. [21] demonstrated that peri-implant sites undergo rapid microbial colonization and exhibit progressive increases in periodontal pathogens, including *F. nucleatum*, during the early stages of healing and function. These observations are consistent with the higher bacterial detection rates and loads observed in the supracrestal implant group in this study. Furthermore, although Kütan et al. [22] primarily focused on radiographic outcomes, their findings highlighted the biological significance of implant placement depth and its influence on peri-implant tissue responses, reinforcing the importance of implant positioning as a factor that affects long-term peri-implant health.

Although bacterial loads increased over time in both groups, significantly greater loads of P. gingivalis and F. nucleatum were observed in the supracrestal implant group at the 12-month evaluation, suggesting that implant positioning may influence long-term peri-implant microbial colonization. From a biological perspective, subcrestal implant placement may provide a more favorable environment for maintaining peri-implant tissue stability. Subcrestal positioning has been associated with improved soft-tissue adaptation, reduced exposure of the implant-abutment interface, and enhanced preservation of crestal bone [23]. These factors may limit the bacterial penetration and reduce the establishment of pathogenic biofilms. The lower bacterial loads and weaker correlations with inflammatory parameters observed in the present study support this concept and suggest a potential microbiological advantage for subcrestal implant placement.

The clinical implications of our findings are significant. Implant positioning should be considered not only from esthetic and prosthetic perspectives but also from a microbiological standpoint. Patients restored with supracrestal implants may require more rigorous maintenance protocols, frequent monitoring, and enhanced plaque control measures to minimize pathogenic bacterial colonization and the risk of peri-implant disease. Quantitative microbial assessment may also be valuable for identifying patients at an increased risk of peri-implant complications.

The present study has certain limitations. The relatively small sample size and single-center design may limit the generalizability of the findings. Although participants were followed prospectively for 12 months, the observational nature of the study precludes definitive conclusions regarding a causal relationship between implant position and peri-implant microbial colonization. Only two bacterial species were evaluated, whereas peri-implant biofilms comprise a complex microbial ecosystem involving numerous pathogenic and commensal microorganisms. Furthermore, a 12-month follow-up may not fully capture long-term changes in peri-implant microbiota and disease progression. Although baseline characteristics were comparable between the groups, the observational cross-sectional design cannot completely eliminate residual confounding from factors such as oral hygiene practices, prosthetic design, implant surface characteristics, occlusal loading, and host-related variables. Furthermore, because implant positioning was determined during routine clinical treatment rather than by random allocation, the absence of randomization limits causal inference and introduces the possibility of selection bias. Future multicenter prospective studies with larger sample sizes, longer follow-up periods, comprehensive microbial profiling using next-generation sequencing, and adjustment for potential confounding variables are warranted to better elucidate the influence of implant position on peri-implant microbiology and long-term implant success.

## Conclusions

Within the limitations of this study, supracrestal implant placement was associated with higher detection rates and significantly greater quantitative levels of *P. gingivalis* and* F. nucleatum* than subcrestal implant placement. Increased bacterial loads in supracrestal implants have also demonstrated stronger correlations with PPD, BOP, and radiographic bone loss. These findings suggest that implant positioning may influence peri-implant microbial colonization and tissue health, with subcrestal placement potentially offering a more favorable microbiological environment and a reduced risk of peri-implant inflammatory complications.

## References

[1] Shyamkumar NT, Patil R, Bhukal S, Mukhopadhyay N, Bhardwaj T, Gupta S (2026). Behavioral determinants of patients' willingness to undergo dental implant therapy: a health belief model-based cross-sectional study. Cureus.

[2] Bentour E, Papamanoli E, Karoussis IK (2025). The decision between tooth retention or replacement with implants: a continuing dilemma. Dent J (Basel).

[3] Banu Raza F, Vijayaragavalu S, Kandasamy R, Krishnaswami V, Kumar VA (2023). Microbiome and the inflammatory pathway in peri-implant health and disease with an updated review on treatment strategies. J Oral Biol Craniofac Res.

[4] Bhukal S, Malik R, Begum R, Adapala K, Killamsetty M, Arora P, Gupta S (2025). Assessing the significance of interleukin-6, interleukin-10, and tumor necrosis factor-alpha in peri-implantitis development: a biomarker study. Cureus.

[5] Chun Giok K, Menon RK (2023). The microbiome of peri-implantitis: a systematic review of next-generation sequencing studies. Antibiotics (Basel).

[6] de Andrade KQ, Almeida-da-Silva CLC, Coutinho-Silva R (2019). Immunological pathways triggered by Porphyromonas gingivalis and Fusobacterium nucleatum: therapeutic possibilities?. Mediators Inflamm.

[7] Pellicer-Chover H, Díaz-Sanchez M, Soto-Peñaloza D, Peñarrocha-Diago MA, Canullo L, Peñarrocha-Oltra D (2019). Impact of crestal and subcrestal implant placement upon changes in marginal peri-implant bone level. A systematic review. Med Oral Patol Oral Cir Bucal.

[8] Galvão TCM, Faria JB de, Borges LH (2022). Microbial diversity in the peri-implant region of patients with history of periodontal disease and clinical parameters. Res Soc Dev.

[9] Löe H (1967). The Gingival Index, the Plaque Index and the Retention Index systems. J Periodontol.

[10] Xiang XM, Liu KZ, Man A, Ghiabi E, Cholakis A, Scott DA (2010). Periodontitis-specific molecular signatures in gingival crevicular fluid. J Periodontal Res.

[11] Evans JS, López-Legentil S, Erwin PM (2018). Comparing two common DNA extraction kits for the characterization of symbiotic microbial communities from Ascidian tissue. Microbes Environ.

[12] Boutaga K, van Winkelhoff AJ, Vandenbroucke-Grauls CM, Savelkoul PH (2003). Comparison of real-time PCR and culture for detection of Porphyromonas gingivalis in subgingival plaque samples. J Clin Microbiol.

[13] Suzuki N, Yoshida A, Nakano Y (2005). Quantitative analysis of multi-species oral biofilms by TaqMan real-time PCR. Clin Med Res.

[14] Yang X, Zhang S, Ning T, Wu J (2025). Fusobacterium nucleatum in health and disease. MedComm (2020).

[15] Canullo L, Peñarrocha-Oltra D, Covani U, Botticelli D, Serino G, Penarrocha M (2016). Clinical and microbiological findings in patients with peri-implantitis: a cross-sectional study. Clin Oral Implants Res.

[16] Romanos GE, Weitz D (2012). Therapy of peri-implant diseases. Where is the evidence?. J Evid Based Dent Pract.

[17] Di Spirito F, Giordano F, Di Palo MP (2024). Microbiota of peri-implant healthy tissues, peri-implant mucositis, and peri-implantitis: a comprehensive review. Microorganisms.

[18] Kumar PS, Mason MR, Brooker MR, O'Brien K (2012). Pyrosequencing reveals unique microbial signatures associated with healthy and failing dental implants. J Clin Periodontol.

[19] Lafaurie GI, Sabogal MA, Castillo DM, Rincón MV, Gómez LA, Lesmes YA, Chambrone L (2017). Microbiome and microbial biofilm profiles of peri-implantitis: a systematic review. J Periodontol.

[20] de Moraes Rego MR, Torres MF, Santiago LC, Lira-Junior R, Lourenço EJ, de Moraes Telles D, Figueredo CM (2015). Osseointegrated implants placed at supracrestal level may harbour higher counts of A. gerencseriae and S. constellatus - a randomized, controlled pilot study. J Oral Microbiol.

[21] Silva-Boghossian CM, Duarte PT, Silva DG, Lourenço TGB, Colombo AP (2023). Colonization dynamics of subgingival microbiota in recently installed dental implants compared to healthy teeth in the same individual: a 6-month prospective observational study. J Appl Oral Sci.

[22] Kütan E, Bolukbasi N, Yildirim-Ondur E, Ozdemir T (2015). Clinical and radiographic evaluation of marginal bone changes around platform-switching implants placed in crestal or subcrestal positions: a randomized controlled clinical trial. Clin Implant Dent Relat Res.

[23] Degidi M, Perrotti V, Shibli JA, Novaes AB, Piattelli A, Iezzi G (2011). Equicrestal and subcrestal dental implants: a histologic and histomorphometric evaluation of nine retrieved human implants. J Periodontol.

